# Bacterial Cellulose-Based Laser-Scribed Graphene Electrode for Hydrogen Peroxide Detection in Cancer Cells

**DOI:** 10.1021/acsabm.5c00825

**Published:** 2025-06-29

**Authors:** Lucas F. de Lima, André L. Ferreira, Letícia Ester dos Santos, Keyla Lívian P. Coelho, Keyla Teixeira Santos, Ariane Schmidt, Marcelo Bispo de Jesus, Thiago R.L.C. Paixão, William R. de Araujo

**Affiliations:** † Departamento de Química Fundamental, Instituto de Química, Universidade de São Paulo, São Paulo, SP 05508-000, Brazil; ‡ Laboratório de Sensores Químicos Portáteis, Departamento de Química Analítica, Instituto de Química, Universidade Estadual de Campinas − UNICAMP, Campinas, SP 13083-861, Brazil; § Nano-Cell Interactions Lab., Departamento de Bioquímica e Biologia Tecidual, Biology Institute, Universidade Estadual de Campinas, Campinas, SP 13083-862, Brazil; ∥ Campinas Electrochemistry Group, Departamento de Físico-Química, 28132 Instituto de Química, Universidade Estadual de Campinas − UNICAMP, Campinas, SP 13083-970, Brazil

**Keywords:** bacterial cellulose, laser-scribed graphene, cancer cells, hydrogen peroxide, natural biopolymer, electrochemical paper-based analytical device

## Abstract

The development of sustainable and high-performance electrochemical sensors is crucial for advancing biomedical applications. In this work, we introduce a hydrogen peroxide (H_2_O_2_) sensor based on bacterial cellulose-derived laser-scribed graphene (BC-LSG), modified with MXene and platinum nanoparticles (PtNPs). Bacterial cellulose (BC), a biodegradable and renewable material, was cultivated and transformed into a highly conductive carbon network using CO_2_ laser irradiation, producing a flexible, portable, and miniaturized electrochemical platform. The incorporation of MXene and PtNPs significantly enhanced the electrocatalytic response toward H_2_O_2_ oxidation, achieving a wide linear concentration range (15–95 μmol L^–1^) and a low detection limit (0.35 μmol L^–1^). Compared to traditional enzymatic sensors, our nanostructured BC-LSG device offers superior stability, reproducibility, and eco-friendliness, aligning with green analytical chemistry principles. The sensor was successfully applied for H_2_O_2_ detection in mammalian cells, demonstrating its potential for real-time monitoring of oxidative stress, a key biomarker in cancer progression and therapeutic responses. This work underscores the synergy between biopolymeric materials, nanotechnology, and laser processing, opening new avenues for scalable, disposable, and sustainable electrochemical devices.

## Graphical abstract

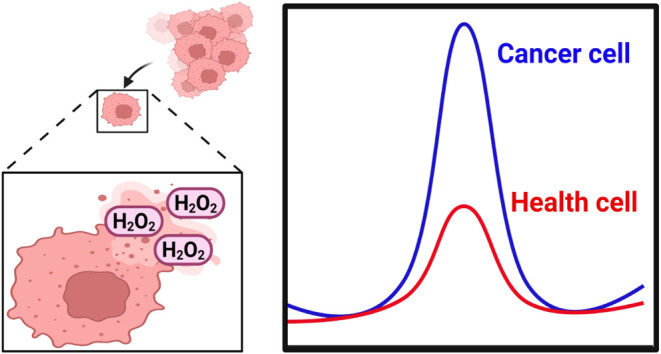

## 1 Introduction

In recent years, the development of advanced materials has been closely coupled with the growing focus on sustainable technologies.^1^ The push for a circular economy is reforming innovation, introducing new perspectives, and redefining traditional approaches. Among the massive group of materials reported worldwide, cellulose stands out as the most abundant and widely used polymer.^2^ Its primary production through plant biomass reaches approximately 125 Gt per year, highlighting its economic significance in industries such as paper manufacturing, biofuel production, and several biomedical applications.^3^ Cellulose can be derived from three main sources such as trees, cotton stalks, and bacterial bioprocesses (bacterial cellulose). Each source yields materials with distinct compositions and physicochemical properties, affecting their suitability for different applications.^3^ Bacterial cellulose (BC) is a highly pure form of cellulose, free from hemicellulose and lignin fibers, which usually are found in plant-derived cellulose that require purification to remove these components.^4^ Additionally, BC consists of a fibrous material (typically 20 μm in length and 50–70 nm in diameter), with excellent mechanical properties suitable for numerous applications.^5^

The bacteria responsible for cellulose production are typically Gram-negative and aerobic, commonly found in fruits, vegetables, and alcoholic beverages.^4^ They require an organic substrate rich in carbon as an energy source to sustain their growth and metabolism.^2^ Hence, several bacterial species can be used to produce BC, including those from the genera , , , and .^6^ These bacterias secrete an extracellular matrix composed of crystalline cellulose, which acts as a protective barrier against UV radiation.^7^ Over time, this process leads to the formation of a solid membrane.

Benefiting from its interesting characteristics, the use of BC in the electronics and sensors field has been explored, contributing to the development of more sustainable and recyclable components.^8,9^ Marketing strategies for electronic devices increasingly prioritize sustainability and flexibility, particularly in applications such as human-machine interfaces, medical monitoring systems, and wearable electronics.^8^ These technologies encompass electronic paper, flexible OLED displays, transistors, and energy storage devices.^10^ A notable processing approach involves the carbonization of BC, which transforms its 3D nanofibrous structure into a highly conductive carbon network, known as carbonized bacterial cellulose (CBC).^11^ This material has demonstrated exceptional potential as an electrode in flexible energy storage devices, such as capacitors, providing full electrolyte accommodation while maintaining excellent mechanical stability under bending and stretching conditions.^12^

The most commonly used method for CBC production involves heating the material in a muffle furnace under a controlled atmosphere and temperature.^11^ This process requires optimal temperature and inert gas conditions (N_2_ or Ar) to obtain high-quality material, suitable for use in electrical or electronic devices. To overcome these limitations, Laser-Scribed Graphene (LSG) has emerged as an alternative technique for producing 3D porous graphene materials with high conductivity.^13^ Contrasting to the conventional methods, LSG does not require precise atmosphere control, masks for conductive track formation, or chemical reagents, allowing for in situ graphene-based material fabrication.^14,15^ Besides, the automation of the technique enables the scalable production of conductive electrodes on various synthetic and natural polymeric substrates.^16–18^

The development of green and sustainable electrodes has become increasingly important as the demand for environmentally responsible technologies grows.^15^ Conventional electrode materials are often derived from fossil-based sources, involve energy-intensive fabrication processes, and contribute to long-term environmental waste.^19^ In contrast, sustainable electrodes, fabricated from renewable, biodegradable, or low-impact materials, offer a more eco-friendly alternative without necessarily compromising performance.^20^ By focusing on green materials, such as cellulose, researchers can reduce the carbon track of device production and support the transition to a more sustainable and circular economy. This approach is particularly relevant in applications like biosensing and wearable electronics, where disposable or short-term use components are common, making biodegradability and environmental compatibility especially valuable.^21^

Our research group has been dedicated to developing various paper-based graphene electrodes for a wide range of applications. De Araujo and coauthors^22^ utilized paperboard to fabricate a conductive material, which was successfully employed in several applications, including clinical, pharmaceutical, food, and forensic analysis. De Lima and collaborators reported the first electrochemical biosensor for human monkeypox detection, using chromatographic paper coated with yellow wax, which was inspired by studies from Fortunato’s group.^23,24^ This substrate was then exposed to CO_2_ laser engraving, resulting in a highly conductive and eco-friendly material. Benefiting from the properties of chromatographic paper for LSG material fabrication, Bottelli and coauthors,^25^ employed chromatography paper coated with a biodegradable polymer to develop a highly conductive material efficient for detecting lidocaine adulterant in cocaine samples. Despite the growing interest in using paper as a substrate for producing 3D porous graphene materials, no reports in the literature have yet explored the use of BC as a substrate for CBC production by the LSG technique. This gap presents an opportunity to investigate BC as a potential candidate for greener analytical applications.

An important type of molecule to be monitored using a CBC material is reactive oxygen species (ROS), including superoxide, peroxide, singlet oxygen, and hydroxyl radicals.^26^ These compounds are key byproducts of oxygen metabolism. These endogenous ROS play a vital role in cell signaling, regulating a wide array of physiological activities and cellular functions.^27^ Among them, H_2_O_2_ stands out as one of the most stable ROS, and it is closely involved in immune responses, pathogen defense, and intracellular signal transduction. Due to its small size and neutral charge, H_2_O_2_ can readily diffuse across cell membranes and reach various organelles.^28^ Elevated levels of H_2_O_2_ have been consistently observed in cancer cells, where it contributes to tumor growth, metastasis, and apoptotic resistance. It also plays a role in enhancing drug responsiveness during targeted therapies, positioning it as a promising biological marker for early stage cancer detection and monitoring.^29^ Despite its diagnostic potential, quantifying H_2_O_2_ in tumor tissues remains a significant challenge due to its low abundance, high reactivity and short lifetime.

Here, we report, for the first time, on the laboratory-scale production of a BC substrate as a precursor for CBC material via the LSG technique. Since paper thickness and grammature are critical factors for the photoconversion of the paper on conductive tracks, we cultivated BC using bacteria at room temperature (25 ± 3 °C). The BC substrate was produced over 30 days, followed by washing and drying steps to obtain high-quality material suitable for graphene-based material production. As a proof-of-concept, our CBC electrode was modified with Ti_3_C_2_T_*x*_ MXene and platinum nanoparticles (PtNPs) for the detection of hydrogen peroxide (H_2_O_2_) generated by mammalian cell lines using linear sweep voltammetry (LSV) technique. Our device successfully detected H_2_O_2_ within a range of 5.0 to 95 μmol L^–1^, demonstrating its suitability for detecting hydrogen peroxide in mouse fibroblast cell lines (3T3), a cell line derived from male hepatoma tissue (HuH-7), and a human breast cancer cell line (MCF-7). The results were consistent with those obtained through fluorescence analysis, demonstrating that our CBC material modified with MXene-PtNPs enables a powerful platform for H_2_O_2_ biomarker detection in healthy and stressed cancer cell lines.

## 2 Experimental Section

### 2.1 Chemicals and Solutions

All reagents used in this work were of analytical grade, and all aqueous solutions were prepared with ultrapure water (Milli-Q SQ 2 Series, Merck) presenting a resistivity of 18.2 MΩ·cm at 25 °C. Sodium tetraborate decahydrate (Na_2_B_4_O_7_·10H_2_O), potassium ferricyanide (K_3_[Fe­(CN)_6_]), potassium ferrocyanide (K_4_[Fe­(CN)_6_]), potassium chloride (KCl), lithium fluoride (LiF), titanium aluminum carbide (Ti_3_AlC_2_), glucose, yeast extract, citric acid, peptone, and sodium monobasic phosphate were purchased from Sigma-Aldrich. Hydrogen peroxide was purchased from Synth. The Ag/AgCl conductive ink used for painting the pseudoreference electrode (RE) and electrical contacts was obtained from Creative Materials (MA, USA).

### 2.2 Apparatus and Characterizations

Cyclic voltammetry (CV), and linear sweep voltammetry (LSV) analyses were performed using an Autolab PGSTAT204 potentiostat/galvanostat (Eco Chemie, Utrecht, Netherlands) controlled by Nova 2.1.4 software. Device fabrication was carried out with a CO_2_ pulsed laser (50 W, Router VS4040C, Visutec, São Paulo, Brazil), following a 2D design created in CorelDraw and processed on a BC substrate. The sensor configuration consisted of a three-electrode system, comprising a working electrode (WE), a counter electrode (CE), and a pseudoreference electrode (RE).

Structural characterization was conducted through Raman spectroscopy using a Horiba T64000 confocal microscope equipped with a 532 nm laser (10 mW power), with an exposure time of 30 s and three accumulation scans. X-ray diffraction (XRD) analysis was performed using a Shimadzu XRD-7000 diffractometer with a Ni filter, employing Cu Kα radiation (λ = 1.54 Å) at a scan speed of 2°/min and a current of 50 mA. Morphological characterization of the CBC, MXene, and CB substrates was carried out via scanning electron microscopy (SEM) using an FEI Quanta FEG 250 Field Emission Gun Scanning Electron Microscope at the LIMicro-IQ Microscopy Core Facility (RRID: SCR 024633).

### 2.3 Synthesis of MXene-PtNP

The synthesis of the MXene nanomaterial followed a previously established protocol.^30,31^ First, 1.0 g of LiF was carefully dissolved in 20 mL of hydrochloric acid (HCl, 6.0 mol L^–1^) at room temperature. Subsequently, 1.0 g of Ti_3_AlC_2_ powder was gradually added to the LiF/HCl solution under vigorous stirring in a Teflon flask, which was maintained in a water bath at 35 °C. The reaction mixture was continuously stirred at this temperature for 24 h to ensure complete etching. After the reaction, the resulting dispersion was subjected to successive washing steps with ultrapure water, followed by centrifugation at 3500 rpm until the supernatant reached a neutral pH. The purified MXene material was then dried at 60 °C and stored in plastic tubes for subsequent use.

Platinum nanoparticles supported on carbon (PtNPs/C) were synthesized using the polyol method. Initially, Vulcan XC72R carbon, sodium polyacrylate (Sigma-Aldrich) and a 3:1 (v/v) ethylene glycol (Sigma-Aldrich) and water mixture were combined in a vessel and sonicated for 20 min to achieve a homogeneous dispersion. Subsequently, an aqueous solution of 2 mmol L^–1^ H_2_PtCl_6_ (Sigma-Aldrich) was added to the dispersion to achieve a 20% (w/w) platinum loading on the catalyst. The mixture was then sonicated for an additional 10 min and heated for 90 s using a household microwave. After cooling to room temperature, the suspension was centrifuged, and the resulting solid was washed thoroughly with Milli-Q water and centrifuged repeatedly five times. Finally, the retained solid was dried at 70 °C for 48 h.

The MXene-PtNPs composite was prepared by mixing 1 mg of MXene in 1 mL of PtNPs dispersion, followed by ultrasonication for 30 min.

### 2.4 Fabrication and Modification of BC-LSG Electrodes

The BC substrates were obtained using (ATCC 23769) following the method described by de Lima and coauthors.^32^ Initially, the was cultured in 1.0 L of Hestrin-Schramm (HS) medium rich in glucose, which had been previously sterilized by autoclaving at 121 °C for 15 min. The inoculated medium was then transferred to a glass container (approximately 40 × 20 cm) and incubated under static conditions at room temperature (25 ± 3 °C) for 30 days, allowing the formation of BC film. After the incubation period, the BC film was carefully collected and subjected to a purification step using a 2.0 mol L^–1^ NaOH solution at 80 °C for 2 h to remove residual bacterial cells and impurities, resulting in a white and flexible substrate. The material was subsequently washed with deionized water until a neutral pH (pH ∼ 7.0). Finally, the purified BC was dried at 60 °C in an oven until complete dehydration, yielding a biodegradable substrate with a thickness of 90.0 ± 1.0 μm.

The CBC-based LSG sensor was fabricated according to the procedure described in the literature.^23,33^ First, the BC substrate was immersed for 30 min in a plastic container containing a 0.1 mol L^–1^ sodium tetraborate solution, followed by a drying step at room temperature overnight. After that, the substrate was subjected to thermal pressing for 30 s at 75 °C, resulting in a flat surface, which was then fixed to a 3 M double-sided sheet (30 cm × 30 cm). The paper substrate was then hydrophobized by spraying with varnish (Goma Laca, from Acrilex) and left to dry at room temperature for 4 h.

The electrochemical sensor on the CB substrate was fabricated using a three-electrode system (1.0 cm × 1.5 cm, with a 3.0 mm diameter working electrode), designed using CorelDraw. To define the geometric electrochemical area, a colorless nail polish from Risqué was used. After optimizing the CO_2_ laser engraving parameters to produce the most conductive tracks (with a laser scan speed of 40 mm s^–1^ and 9.5% laser power (from 50 W)), the pseudoreference electrodes and electrical contacts were painted with Ag/AgCl conductive ink and cured at 70 °C for 15 min. Lastly, 0.5 μL of MXene-PtNPs were dropped on the working electrode and dried at room temperature to be used for H_2_O_2_ detection.

### 2.5 Electrochemical Measurements

The analytical curve for H_2_O_2_ detection ranging from 15 to 95 μmol L^–1^ was obtained using the BC-LSG/MXene-PtNP sensor and LSV technique at a scan rate of 50 mV s^–1^ and a potential window ranging from 0.0 to 0.75 V in 0.1 mol L^–1^ KCl. Reproducibility studies were performed with a 45 μmol L^–1^ H_2_O_2_ solution in 0.1 mol L^–1^ KCl, using 10 different manufactured electrodes. Selectivity studies were conducted to assess potential interfering compounds commonly found in cell culture media, such as dopamine, uric acid, glucose, citric acid, and lactate. These compounds were tested both individually and in a mix with H_2_O_2_ at a 1:1 ratio (H_2_O_2_:interferent) at a concentration of 45 μmol L^–1^.

### 2.6 Cell Culture

Huh7, MCF-7 and 3T3 cell lines were maintained in a Panasonic incubator at 37 °C with 95% relative humidity and 5% CO_2_. Cells were cultured in Dulbecco’s modified Eagle’s medium (DMEM; Life Technologies, Canada) supplemented with 10% fetal bovine serum (FBS; Gibco, South America) and 1% penicillin–streptomycin (Pen/Strep; Gibco, USA). MCF-7 cells were maintained in Roswell Park Memorial Institute (RPMI) medium (Life Technologies, Canada) with the same supplementation. Subculture was performed as needed to maintain approximately 80% confluence. To verify the absence of mycoplasma contamination, cells were cultured without antibiotics for 3 days and tested using the direct DNA staining method with Hoechst 33342 (Invitrogen, USA), followed by fluorescence microscopy analysis.

### 2.7 Detection of Cellular ROS through Fluorescent Assay

For reactive oxygen species (ROS) detection, cells were plated in 96-well plates at a density of 2 × 10^5^ cells/well in a supplemented medium and incubated at 37 °C with 5% CO_2_ for 24 h to allow adhesion. After this period, cells incubated with Phorbol 12-myristate 13-acetate (PMA, 20 mmol L^–1^) in Hank’s Balanced Salt Solution (HBSS, Gibco, USA), which served as a positive control, while cells incubated with HBSS alone served as the negative control. Following 2 h of incubation, the medium was replaced with CM-H2DCFH-DA, (Invitrogen, USA) solution (100 μL, 5 m mol L^–1^ in HBSS) and cells were further incubated for 30 min at 37 °C under controlled atmospheric conditions. Excess, noninternalized DCFH-DA or its oxidized product (DCF) was removed from the extracellular medium by gently washing the cells twice with 1× PBS (100 μL). After washing, fluorescence intensity was measured using the Cytation 5 Hybrid Multi-Detection Reader (BioTek Instruments, Winooski, VT, USA), hereafter referred to as Cytation 5, with an excitation wavelength of 485 nm and an emission wavelength of 528 nm. Fluorescence intensity was expressed as a percentage relative to untreated control wells. Data were analyzed using GraphPad Prism 8.0.2, and statistical significance was determined by Student’s *t* test, with *p* < 0.05 considered significant.

Fluorescence images were acquired at 12 sites per well using a 10× objective on the Cytation 5. Imaging was acquired using the following filter cubes: the GFP filter cube (EX 469/35 nm, EM 525/39 nm, dichroic mirror 497 nm, LED 465 nm) for DCF and the DAPI filter cube (EX 377/50 nm, EM 447/60 nm, dichroic mirror 409 nm, LED 365 nm) for Hoechst 33342. Fluorescence images were acquired under identical exposure settings across all conditions to ensure comparability.

## 3 Results and Discussion

### 3.1 Fabrication and Optimization of BC-LSG Electrode

To obtain a BC sheet with adequate thickness, was incubated in the HS medium with a high glucose concentration for 30 days at room temperature (Figure 1A**)**, resulting in a thick substrate suitable for our study. The BC substrate is formed due to the protective mechanism by , leading to a homogeneous material produced by multiple bacterial colonies. Figure 1B shows SEM images of the BC substrate after 30 days of incubation, without prior cleaning. The micrographs, obtained at magnifications of 5,000×, 10,000×, and 20,000×, show numerous rod-shaped bacteria dispersed on the fibrous BC membrane. The treated BC substrate was used as a flexible and stable platform for the photoconversion of cellulosic material to conductive tracks, which are desired for electrochemical applications.

**Figure 1:**
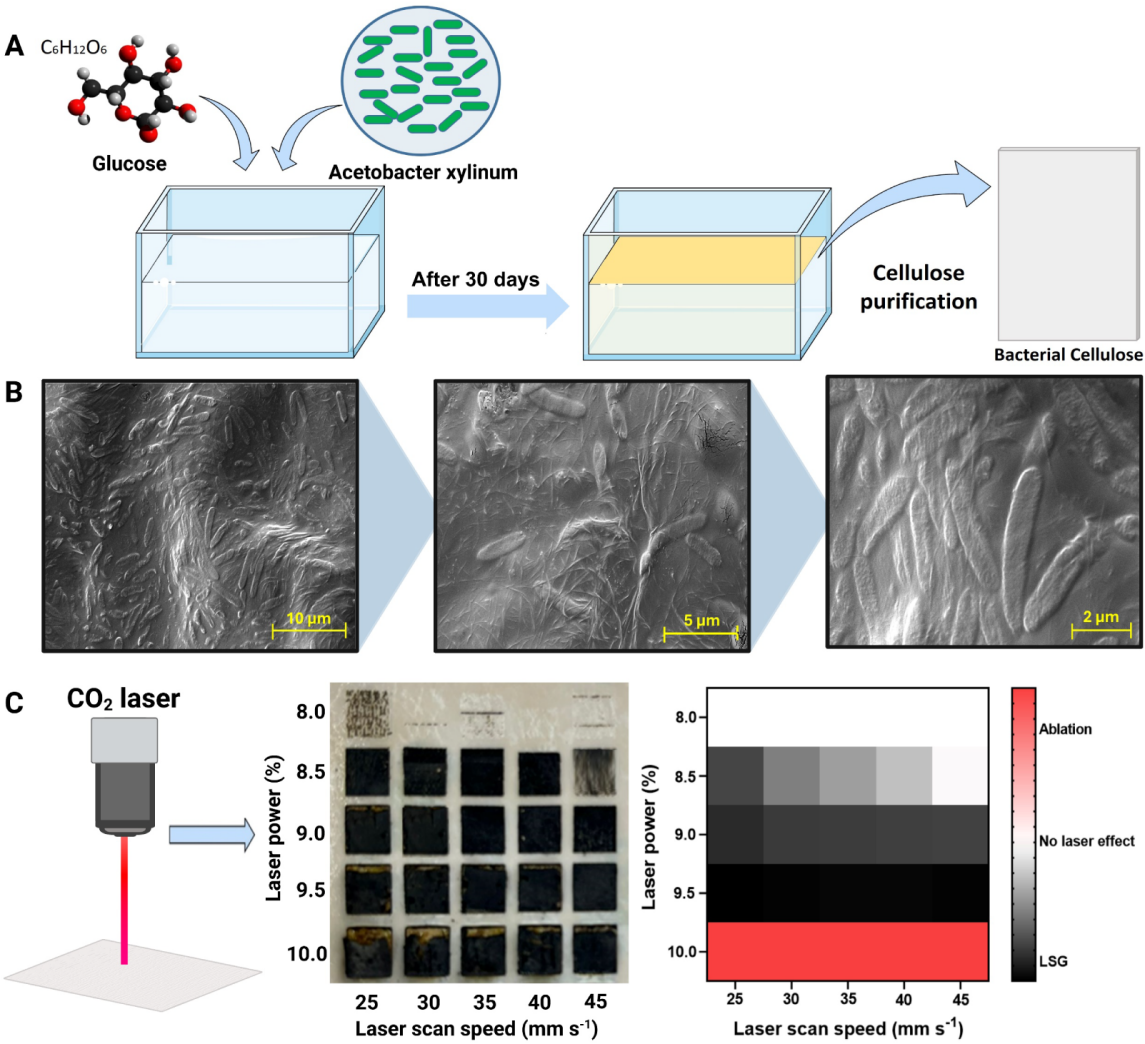
(A) Schematic representation of BC production using cultured in a glucose-rich medium for 30 days, followed by purification to obtain a BC membrane. (B) SEM images of the BC substrate before purification. Rod-shaped bacteria fixed within the fibrous cellulose matrix is observed. The images were captured at magnifications of 10,000×, 20,000×, and 50,000×. (C) Optimization of CO_2_ laser parameters for the in situ conversion of BC into CBC. The left panel shows macroscopic images of laser-treated BC at different laser power (8.0–10.0%; from 50 W) and laser scan speed (25–45 mm s^–1^) conditions. The right panel presents a heatmap summarizing the effects of laser parameters on the formation of LSG, highlighting the optimal conditions (black region) for obtaining a conductive structure while avoiding material ablation (red region) or insufficient laser effects (white region).

The controlled production of an optimal conductive material on a paper substrate depends on several parameters, such as the type of paper, cellulosic fiber composition, and grammature.^34,35^ To prevent the complete ablation of the material by the CO_2_ laser radiation, the BC substrate was treated with sodium tetraborate, a known fire retardant.^35^ In addition, the parameters and conditions for using CO_2_ laser engraving to produce in situ CBC-based LSG electrodes have not been previously reported in the literature. Therefore, they were evaluated in this work to generate high-performance electrodes, as the optimization of these parameters plays a crucial role in device performance. Therefore, laser power and laser scan speed were analyzed to achieve an optimal BC-based LSG electrochemical sensor (Figure 1C). We evaluated laser power values ranging from 8.0% to 10% (from 50 W) and laser scan speeds from 25 to 45 mm s^–1^. The electrical resistance of the CBC-based LSG material was measured using a digital multimeter, demonstrating that a laser power of 9.5% (from 50 W) produced the most conductive material across all evaluated laser scan speeds.

In our study, the CB-LSG exhibits comparable electrochemical performance to PI-LSG electrodes. Specifically, the cellulose-based LSG shows lower charge transfer resistance (Rct) and a more pronounced capacitive behavior, which we attribute to its more porous structure and higher surface area. Additionally, the sustainable and biodegradable nature of cellulose offers a significant environmental advantage over synthetic PI substrates.

Although PI-LSG is known for its high conductivity and mechanical stability, our cellulose-based material achieves similar conductivity values while offering a greener and potentially more cost-effective alternative. These findings suggest that cellulose-derived LSG can be a promising alternative for conventional PI-LSG in various sensing applications, especially where sustainability is a priority

### 3.2 Morphological, Structural, and Electrochemical Characterizations

#### 3.2.1 BC-LSG Electrode Characterizations

To obtain additional information on the optimal parameters for CO_2_ laser engraving on BC substrate, we fixed the laser power at 9.5% (the optimal condition) and fabricated several electrodes using different laser scan speeds (ranging from 25 to 45 mms^–1^). Sheet resistance, Raman spectroscopy, and CV measurements were performed under each condition to assess both structural, electric and electrochemical performance. First, we measured the sheet resistance for each condition, considering the area and thickness of the material. As shown in Figure 2A, we observed variations in sheet resistance and visible graphitization on the BC substrate. At a laser scan speed of 40 mm s^–1^ and a laser power of 9.5%, we achieved the most reproducible results and the lowest sheet resistance, which is in agreement with the sheet resistance values obtained for chromatographic paper substrate reported in the literature.^36^

**Figure 2:**
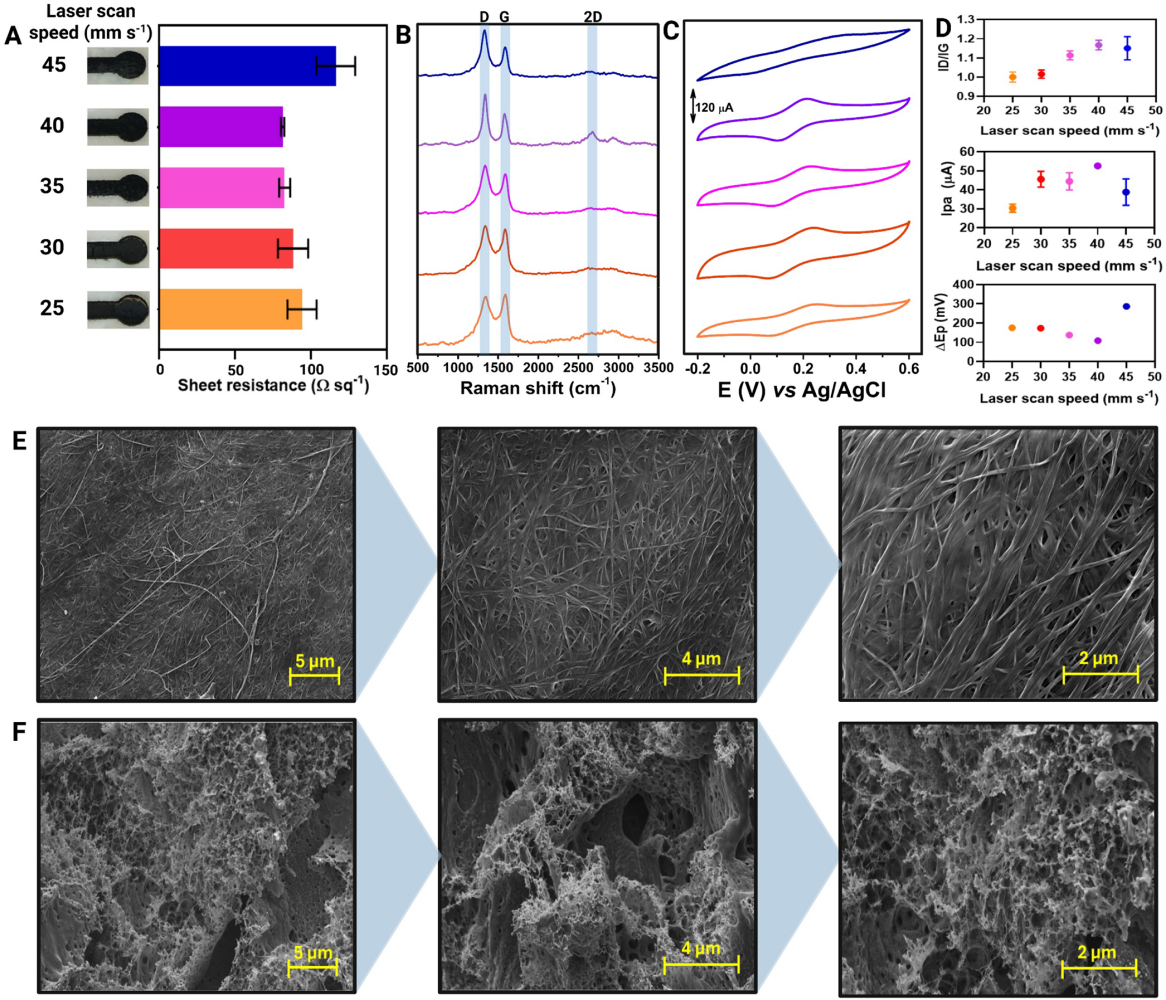
Influence of laser scan speed on the structural, electrical, and electrochemical properties of BC-LSG electrode. (A) Sheet resistance measurements for LSG produced at different laser scan speeds (25–45 mm s^–1^). (B) Raman spectra of the LSG sensors, highlighting characteristic D, and G bands, with variations in intensity related to laser processing conditions. (C) CV plots of LSG electrodes recorded in the presence of 5.0 mmol L^–1^ [Fe­(CN)_6_]^3–/4–^ in 0.1 mol L^–1^ KCl, demonstrating the electrochemical performance at different laser scan speeds. (D) Summary of main electrochemical and spectroscopy parameters, including the intensity ratio of the D and G bands (ID/IG), Ipa, and ΔEp, as a function of laser scan speed. (E) SEM images of untreated BC, showing its fibrous and highly porous structure at different magnifications (6,000×, 13,000×, and 25,000×). (F) SEM images of LSG, revealing the morphological transformation of BC into a graphitic, porous structure after laser treatment at different magnifications (6,000×, 13,000×, and 25,000×).

Raman spectroscopy was used to evaluate the structural effects under each laser scan speed condition (Figure 2B). The main characteristic bands for carbon materials were analyzed: the G band, which is attributed to in-plane vibrations of the sp^2^-hybridized graphitic carbon domains, the D band, which is the breathing mode of carbon rings and arises by the presence of defects, such as the nongraphitic (sp^3^) carbon, and the D+G band (or 2D) is assigned to the structural organization of sp^2^ carbon. These bands emerged at 1577 ± 2 cm^–1^, 1339 ± 2 cm^–1^, and ∼2660 cm^–1^, respectively.^37–40^ To obtain further insights into the electrochemical performance of our BC-LSG electrode, we performed CV measurements in the presence of 5.0 mmol L^–1^ [Fe­(CN)_6_]^3–/4–^ in 0.1 mol L^–1^ KCl, using a scan rate of 50 mV s^–1^ over a potential window ranging from −0.2 to 0.6 V. The measurements resulted in a quasi-reversible behavior of the CV plots for all evaluated conditions^41^ (Figure 2C), likely due to the uncompensated resistance of the electrode materials generated.

To obtain additional insights from Raman spectroscopy and CV plots, we calculated the main parameters for each technique under different conditions and compared the results (Figure 2D). First, the degree of disorder was assessed using the ID/IG ratio. As the laser scan speed increased from 25 to 45 mm s^–1^, the disorder degree also increased, ranging from 1.00 ± 0.01 at 25 mm s^–1^ to 1.15 ± 0.05 at 45 mm s^–1^ (*n* = 3), in agreement with the observed in the normalized Raman spectra of Figure 2B. Interestingly, the highest value was observed at 40 mm s^–1^ (1.17 ± 0.02), which also corresponded to the lowest sheet resistance value. The 2D band intensity increased from 25 to 45 mm s^–1^, with the highest intensity at 40 mm s^–1^, indicating higher crystallinity. To assess the electrochemical performance from CV plots, the anodic peak current (Ipa) and peak-to-peak potential separation (ΔEp) were extracted and compared across all evaluated conditions.

The first parameter evaluated was the anodic peak current (Ipa), keeping the laser power fixed at 9.5% and changing the laser scan speed from 25 to 45 mm s^–1^. The Ipa increased from 25 to 40 mm s^–1^, supplemented by a decrease in ΔEp within the same laser power range, which is in line with the sheet resistance tendency of the BC-LSG material, as expected. Since higher laser scan speeds (>40 mm s^–1^) did not further enhance electrochemical performance, enabling the choice of 40 mm s^–1^ with the best parameter condition. Under optimized conditions (laser power of 9.5% and laser scan speed of 40 mm s^–1^), the LSG method produced a CBC-based LSG material with an Ipa of 52.6 ± 0.6 μA (*n* = 3) and an ΔEp of 88.7 ± 4.5 mV (*n* = 3) for the [Fe­(CN)_6_]^3––/4–^ redox probe. These results demonstrate that our BC-LSG sensor exhibits excellent structural and electrochemical characteristics, further highlighting its high performance.

SEM images were obtained for the BC substrate before and after CO_2_ laser engraving (Figure 2E,F). The chemically treated BC substrate exhibited long and continuous fibers, characteristic of BC material.^4,42,43^ After engraving under optimized laser conditions, the CBC-based LSG electrode showed a highly porous structure, with an interconnected graphene-based network, enhancing the electrode’s surface area. Figure S1 shows the design of the device under optimized conditions

#### 3.2.2 BC-LSG/MXene-PtNP Characterizations

Once the BC-LSG electrode was characterized, the synthesized MXene/PtNPs composite was drop-casted on the BC-LSG working electrode. Figure 3A presents a schematic representation of the Ti_3_C_2_T_*x*_ MXene structure before and after the etching, showing the presence of the functional groups at the surface. Figure 3B presents the SEM image of the synthesized MXene and its characteristic layered morphology, as expected for this material.^44^ The XRD in Figure 3C compares the precursor MAX phase Ti_3_AlC_2_ (in red) and the synthesized Ti_3_C_2_T_*x*_ MXene (in black) after the etching with LiF and HCl. The appearance of only the (002) and (004) peaks of MXene can be attributed to the sample preparation without pressing it to orient the flakes to the (00l) direction.^45^ The XRD pattern of the PtNPs/C is presented in Figure S2. The (002) carbon plane and the (111), (200), (220), (311), and (222) planes of Pt are identified in the graph. The larger Pt peak suggests the successful formation of the nanoparticles (NPs).^46^ These results are in agreement with the expected crystallographic features for these nanomaterials.

**Figure 3:**
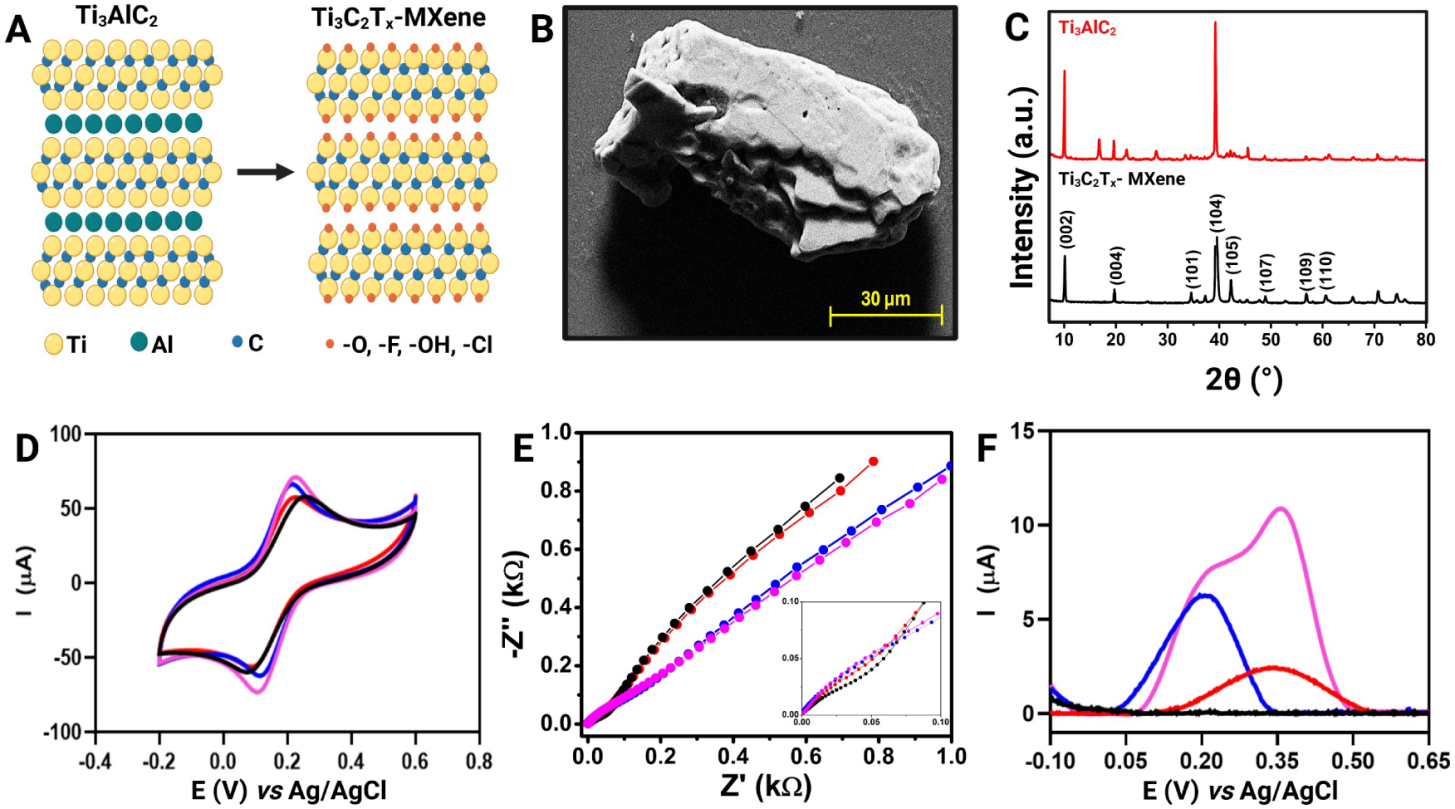
(A) Scheme of MXene synthesis from etching the Al with LiF/HCl. (B) SEM micrograph of the MXene after synthesis. (C) X-ray diffractogram of the precursor Ti_3_AlC_2_ (in red) and the Ti_3_C_2_T_X_ MXene after synthesis (in black) with the planes assigned in the graph. (D) CVs, (E) EIS, and (F) baseline-corrected LSV of the BC-LSG (black line), MXene-modified BC-LSG (red line), PtNPs/C-modified BC-LSG (blue line), and MXene/PtNPs/C-modified BC-LSG electrode (pink line). All the experiments were conducted in triplicate (*n* = 3). The CV and EIS measurements were carried out in the presence of 5.0 mmol L^–1^ [Fe­(CN)_6_]^3–/4–^ in 0.1 mol L^–1^ KCl. LSV measurements were performed in the presence of 45 μmol L^–1^ H_2_O_2_ in 0.1 mol L^–1^ KCl. All voltammetric measurements were performed using a scan rate of 50 mV s^–1^.

To obtain additional information about the performance of the MXene-PtNP on BC-LSG electrode, CV, EIS and LSV measurements were performed. According to the CV and EIS plots (Figure 3D,E) carried out in the presence of 5.0 mmol L^–1^ [Fe­(CN)_6_]^3–/4–^ in 0.1 mol L^–1^ KCl, no significant difference between the nonmodified and modified electrodes was observed, which can be explained due to the lower amount of MXene-PtNP optimized to detect H_2_O_2_ (Figure S3). Also, the CV plots shows a peak-to-peak potential separation between 65–82 mV, indicating a quasi-reversible redox reaction with fast kinetics (Figure 3D). As the kinetics become more favorable, the electrochemical process becomes limited by mass transfer, which is reflected in the Nyquist plot by the absence of a well-defined semicircular region (Figure 3E).^47^ However, the LSV plots indicate that BC-LSG alone does not exhibit high detectability for H_2_O_2_ (black line). After modification with MXene nanomaterial, an oxidation peak becomes visible close to 0.35 V (red line). Meanwhile, the PtNP-modified BC-LSG presented electrocatalytic oxidation of H_2_O_2_, with a peak current around 0.2 V. Lastly, the synergistic effect between MXene and PtNP enhances the oxidation peak current for H_2_O_2_ detection, enabling an increase in sensitivity and detectability (Figure 3F).

### 3.3 Electroanalytical Performance of the BC-LSG/MXene-PtNP Sensor

The optimized BC-LSG/MXene-PtNPs device was used for the electroanalytical detection of H_2_O_2_ using the optimal amount of composite nanomaterial (MXene-PtNPs) (Figure S2). LSV was employed, and the Ipa values were extracted from baseline-corrected LSV plots in the presence of H_2_O_2_. In mammalian cells, H_2_O_2_ is a major reactive oxygen species (ROS) generated as a metabolic product, primarily in mitochondria (Figure 3A). It plays an important role in cell signaling, immune responses, and redox homeostasis, and its excessive accumulation can lead to oxidative stress, damaging biomolecules such as proteins, lipids, and DNA.^26,48,49^

Under optimized laser engraving and nanomaterial modification conditions, the BC-LSG/MXene-PtNPs sensor demonstrated high sensitivity for H_2_O_2_ detection within seconds. LSV plots (Figure 3B) showed a linear response to H_2_O_2_ concentrations ranging from 15 to 95 μmol L^–1^ in 0.1 mol L^–1^ KCl, with a determination coefficient (R^2^) of 0.996 (Figure 3C). The limit of detection (LOD) and limit of quantification (LOQ) were calculated following the IUPAC method,^50^ resulting in an LOD of 0.35 and an LOQ of 1.16 μmol L^–1^, confirming the sensor’s excellent analytical performance. Also, the reproducibility of our modified BC-LSG sensor was evaluated using 10 different devices (*n* = 10) in the presence of 45 μmol L^–1^ of H_2_O_2_. The sensor exhibited a relative standard deviation (RSD) of 4.84%, indicating high reproducibility and consistency of the fabrication process. These results confirm the reliability of our method for H_2_O_2_ detection.

One of the main advantages of our sensor is its environmentally friendly nature, which can be quantitatively assessed using the Analytical GREEnness Metric (AGREE) approach.^51^ This method evaluates the sustainability of analytical procedures and devices by considering their environmental impact and safety for human health. Figure 4E presents the AGREE assessment results, based on the 12 principles of green analytical chemistry, standardized on a scale from 0 to 1. Our sensor achieved a score of 0.84 (green color), indicating a high level of environmental sustainability. This result is attributed to the miniaturized design, which reduces energy consumption, reagent usage, and waste generation. Additionally, the use of BC-based substrates and LSG fabrication eliminates the need for solvents or hazardous chemicals, further enhancing the eco-friendly profile of our method.

**Figure 4:**
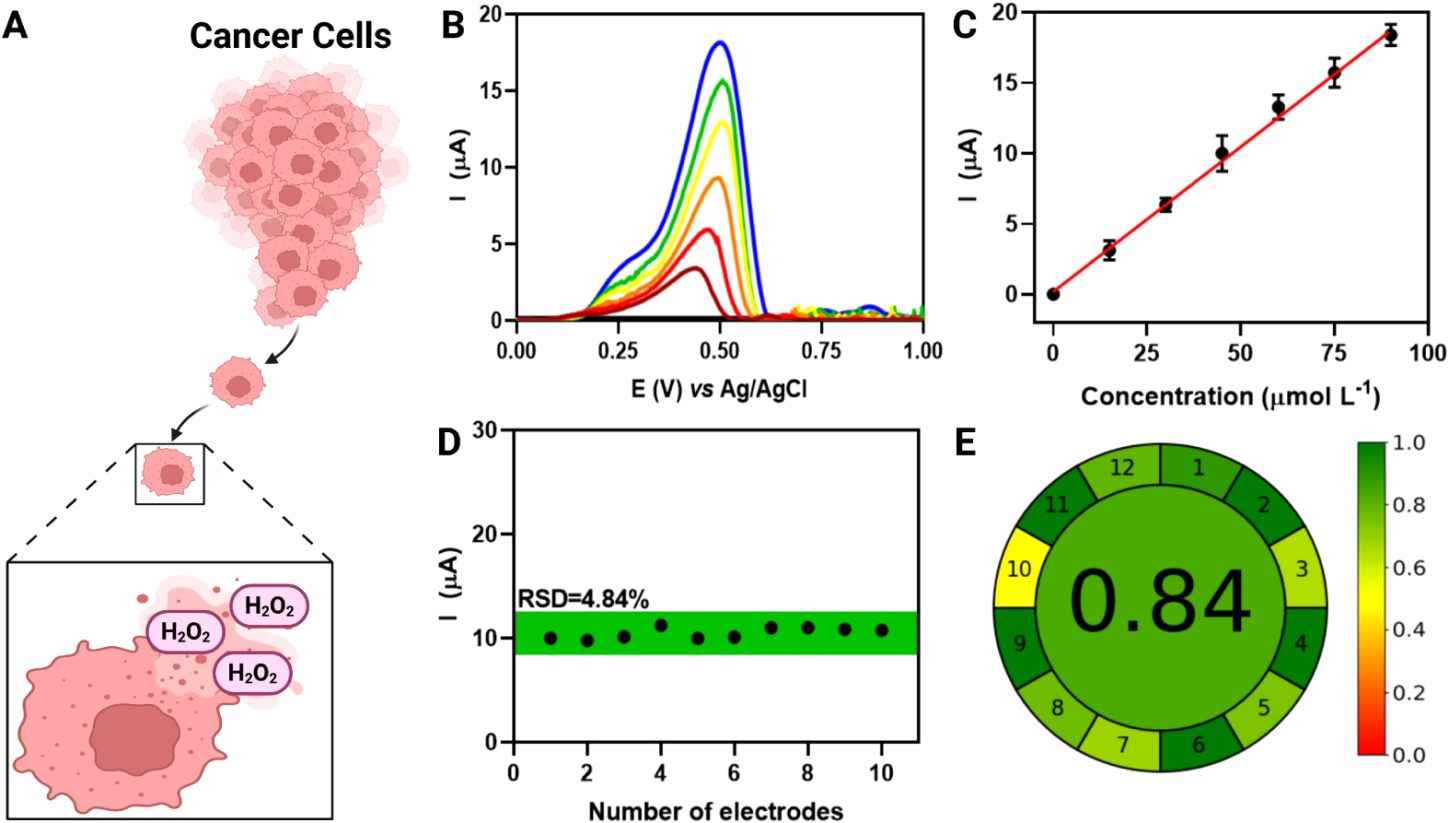
(A) Schematic representation of H_2_O_2_ production by cancer cells, highlighting its role as a metabolic subproduct. (B) Baseline-corrected LSV curves obtained for different concentrations of H_2_O_2_, showing an increase in anodic peak current with increasing concentrations. (C) Calibration curve of the BC-LSG/MXene-PtNPs sensor for H_2_O_2_ detection, demonstrating a linear response in the concentration range of 15 to 95 μmol L^–1^ (R^2^ = 0.996). (D) Reproducibility assessment of the sensor using 10 different electrodes (*n* = 10) at 45 μmol L^–1^ H_2_O_2_, yielding a relative standard deviation (RSD) of 4.84%. (E) AGREE score obtained for the modified BC-LSG sensor, with a score value of 0.84, confirming the high eco-friendly and sustainability characteristics of our sensor.

Collectively, the combination of high analytical performance and environmental sustainability makes our sensor a promising alternative for H_2_O_2_ detection in cancer cells. Its suitability for decentralized testing analyses highlights its potential for advancing the development of disposable and eco-friendly devices.

Compared to other sensors reported in the literature for H_2_O_2_ detection (Table S1), our modified CBC-based LSG sensor demonstrates comparable detectability and a broader linear response range. Additionally, the use of LSV enables rapid detection within 10 s, along with a low limit of detection (LOD) and limit of quantification (LOQ) for H_2_O_2_ oxidation. The sensor combines advantageous characteristics such as being disposable, miniaturized, and portable, with an environmentally friendly fabrication process, underscoring its potential for advancing paper-based electrochemical devices based on biopolymeric materials. The selectivity of our device was evaluated in the presence of common species found in the cell culture medium, such as dopamine, uric acid, glucose, citric acid, and lactate. These compounds were tested both individually and in a mix with H_2_O_2_ at a 1:1 ratio (H_2_O_2_:interferent) at a concentration of 45 μmol L^–1^ (Figure S4). According to the selectivity studies, no interference was observed in the presence of the evaluated molecules, demonstrating the high selectivity of our BC-LSG/MXene-PtNPs sensor toward H_2_O_2_ sensing.

### 3.4 Electrochemical Measurements of H_2_O_2_ in Health and Cancer Cells

The intensified generation of reactive oxygen species (ROS), including hydrogen peroxide (H_2_O_2_), is a well-established metabolic characteristic of tumorigenic cancer cells when compared to healthy cells, leading to redox imbalance and directly affecting the antitumor immune response.^27^ Therefore, in this study, we aimed to assess ROS (H_2_O_2_) production in different cell lines by comparing the electrochemical BC-LSG/MXene-PtNPs sensor with the standard fluorescence-based quantification method (DCFH-DA).

Huh7 and MCF-7 exhibit higher hydrogen peroxide production compared to nontumor 3T3 cells, both under basal conditions and upon oxidative stress induction with PMA (Figure 5). Electrochemical analysis revealed that the recorded currents were significantly higher in the cancer cell lines Huh7 and MCF-7 under stress induction, while 3T3 cells exhibited a modest increase (Figure 5A). These findings align with the fluorescence quantification results (Figure 5B), where ROS signal intensity was markedly higher in cancer cells under oxidative stress compared to 3T3 cells. This difference is visually evident in the stronger green fluorescence signal in Huh7 and MCF-7 cells treated with PMA (Figure 5C). These results suggest that cancer cells generate more H_2_O_2_, likely due to intensified metabolism and mitochondrial dysfunction—hallmarks of tumorigenic cells.^52^

**Figure 5:**
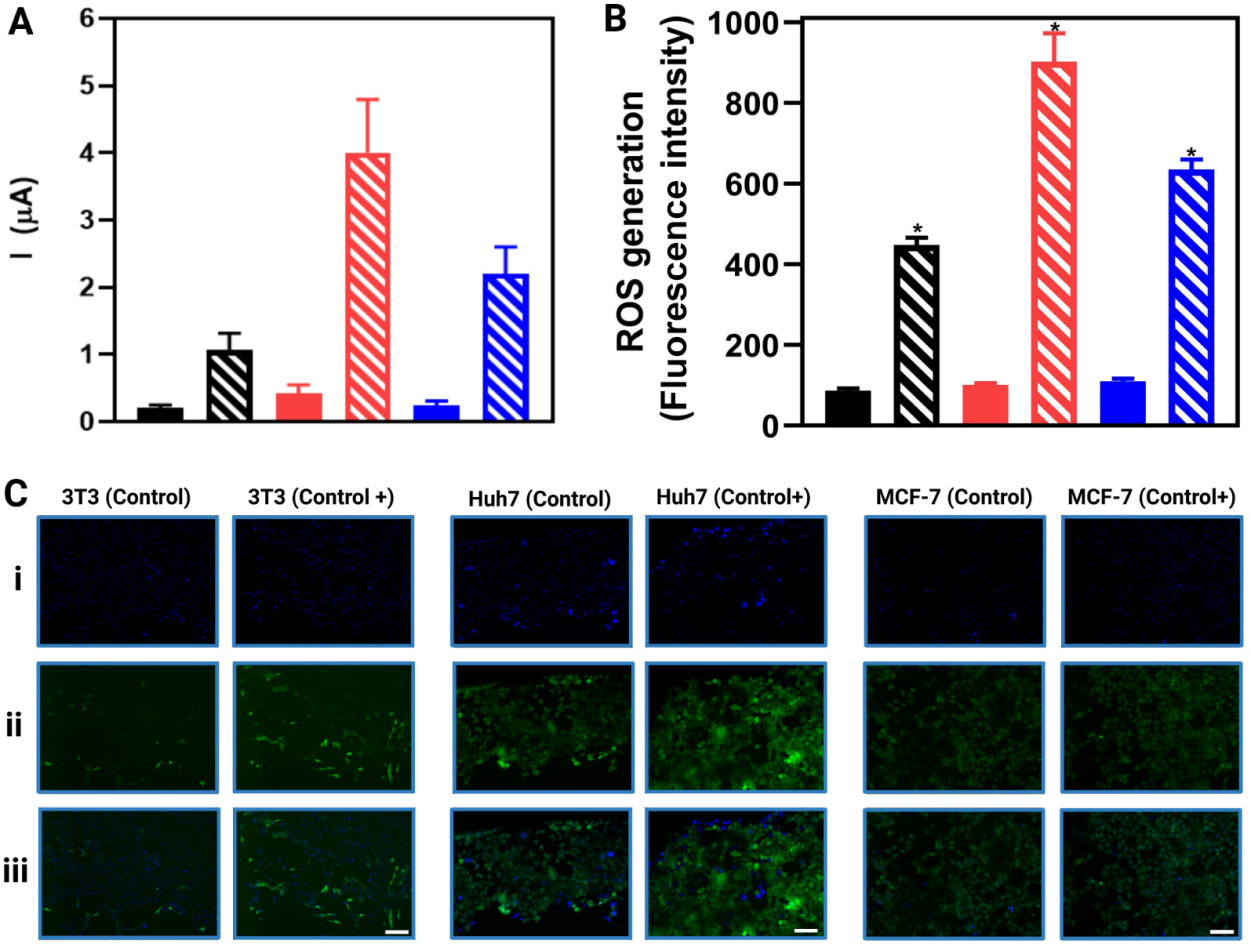
Detection of reactive oxygen species (ROS) and hydrogen peroxide in nontumor (3T3 (black bars)) and cancer (Huh7 (red bars) and MCF-7 (blue bars)) cells under normal and oxidative stress conditions. (A) Electrochemical response obtained using a BC-LSG/MXene-PtNPs sensor, highlighting hydrogen peroxide generation in different cell lines. (B) Quantification of ROS by fluorescence, indicating the relative intensity of reactive oxygen species production in the analyzed cells. Solid bars represent cells without induced stress, while striped bars indicate cells treated with PMA (20 mmol L^–1^) to induce ROS production. (C) Fluorescence microscopy of 3T3, Huh7, and MCF-7 cells without stress induction (columns “Control”) and after ROS induction by PMA (columns “Control+”). Images (i) represent nuclear staining (blue), while (ii) and (iii) show ROS labeling (green), revealing a significant increase in fluorescence in cancerous cells treated with PMA. The scale bars correspond to 200 μm. The quantification of reactive oxygen species (ROS) was performed by fluorescence, allowing the assessment of the relative intensity of ROS production in the analyzed cells. Solid bars represent cells without induced stress, while striped bars indicate cells treated with PMA (20 mmol L^–1^) to induce ROS production. For statistical analysis, a two-way ANOVA was applied to evaluate the effects of cell type and treatment. The fill bars corresponding to the control and the sparse bars corresponding to the positive control. The test revealed that both factors significantly influenced the cellular response (*p* < 0.05). Additionally, a significant interaction between factors was observed (*p* < 0.05), indicating that ROS induction by PMA varied among different cell lines.

The electrochemical BC-LSG/MXene-PtNPs sensor showed a strong correlation with traditional image-based ROS quantification, highlighting its potential for cellular analysis applications. Additionally, the differential response observed between normal and nontumor cells underscores the sensor’s capability to discriminate cellular redox states, offering promising applications for monitoring redox-based therapies and screening antioxidant compounds.

## 4 Conclusion

Our results demonstrate the viability of fabricating LSG electrodes on BC substrate, as an alternative and sustainable route for disposable sensing devices. The BC-LSG/MXene-PtNP sensor exhibits remarkable sensitivity, selectivity, and reproducibility for H_2_O_2_ detection. The synergistic effect of MXene and PtNPs significantly enhanced the electrochemical performance, enabling a broad linear detection range and a lower detection limit. Moreover, its applications to sensing H_2_O_2_ in mammalian cells highlight the sensor’s potential for studying oxidative stress-related pathologies, including cancer.

Beyond its analytical performance, the proposed sensor aligns with green analytical chemistry principles, offering a cost-effective and scalable alternative to conventional sensors. This work paves the way for future advancements in bioanalytical devices, particularly in the development of wearable and disposable electrochemical sensors for personalized medicine. By integrating sustainable materials with state-of-the-art nanotechnology, our approach contributes to the next generation of miniaturized, high-performance diagnostic tools with real-world biomedical applications.

## Supplementary Material



## Notes

Digital images of the electrochemical sensor, XRD spectra of the PtNPs, optimization of the PtNP-MXene amount on the working electrode (WE), interference study, and a table comparing this work with others reported in the literature (PDF)
