# Supplementary material for: Bacterial Cellulose-Based Laser-Scribed Graphene Electrode for Hydrogen Peroxide Detection in Cancer Cells
Source: ACS Appl Bio Mater. 2025 Jun 29;8(7):6339–49. doi: 10.1021/acsabm.5c00825 (PMC12284866; doi:10.1021/acsabm.5c00825)
Supplement: Supplementary file 1 [file mt5c00825_si_001.pdf]

SUPPORTING INFORMATION

A Bacterial Cellulose-Based Laser-Scribed Graphene Electrodes Toward Hydrogen Peroxide Detection in Cancer Cells

Lucas F. de Lima[a,b*], André L. Ferreira[c], Letícia Ester dos Santos[b,c], Keyla Lívian P. Coelho[c], Keyla Teixeira Santos[d], Ariane Schmidt[d], Marcelo Bispo de Jesus[c], Thiago R.L.C. Paixão[a*] and William R. de Araujo[b*].

[a] Departamento de Química Fundamental, Instituto de Química, Universidade de São Paulo, São Paulo, SP 05508-000, Brasil.

[b] Laboratório de Sensores Químicos Portáteis, Departamento de Química Analítica, Instituto de Química, Universidade Estadual de Campinas – UNICAMP, 13083-861, Campinas, SP, Brazil.

[c] Nano-Cell Interactions Lab., Departamento de Bioquímica e Biologia Tecidual, Biology Institute, Universidade Estadual de Campinas, 13083-862, Campinas, SP, Brasil.

[d] Campinas Electrochemistry Group, Departamento de Físico-Química, Instituto de Química, Universidade Estadual de Campinas – UNICAMP, 13083-970, Campinas, SP, Brazil.

*Corresponding author:

E-mail address: delimalf@unicamp.br (Lucas Felipe de Lima). trlcp@iq.usp.br (Thiago R.L.C. Paixão) and, wra@unicamp.br (William Reis de Araujo)

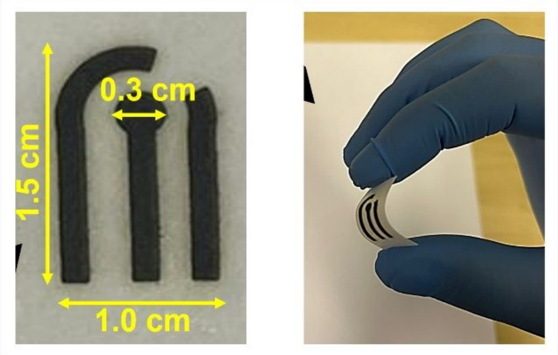

Figure S1: Electrode design produced under optimized conditions using CO_2 laser engraving.

Supporting Information 1: X-Ray diffraction (XRD) PtNPs characterization

XRD patterns were obtained using a Bruker D2 Phaser diffractometer, equipped with a monochromatic CuKa radiation source and a Ni filter. Data acquisition was performed with a LYNXEYE™ linear detector, using continuous scanning with a step size of 0.02° 2θ over the range of 10° to 90°, and a scan speed of 0.3 seconds per step.

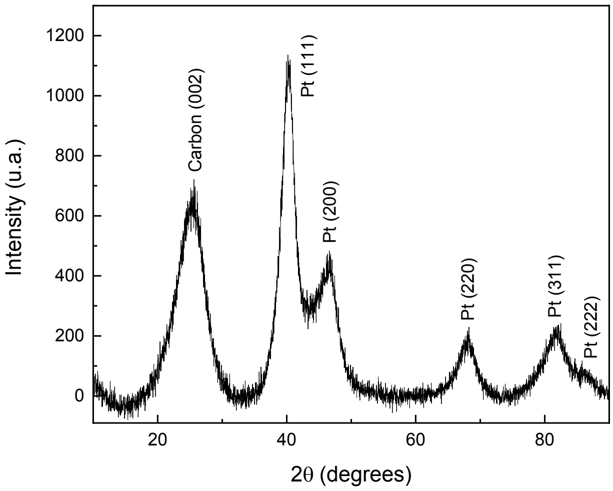

Figure S2: X-ray diffraction (XRD) pattern of (PtNPs/C). The specific angles associated with the crystal planes of both carbon and platinum are annotated in the figure.

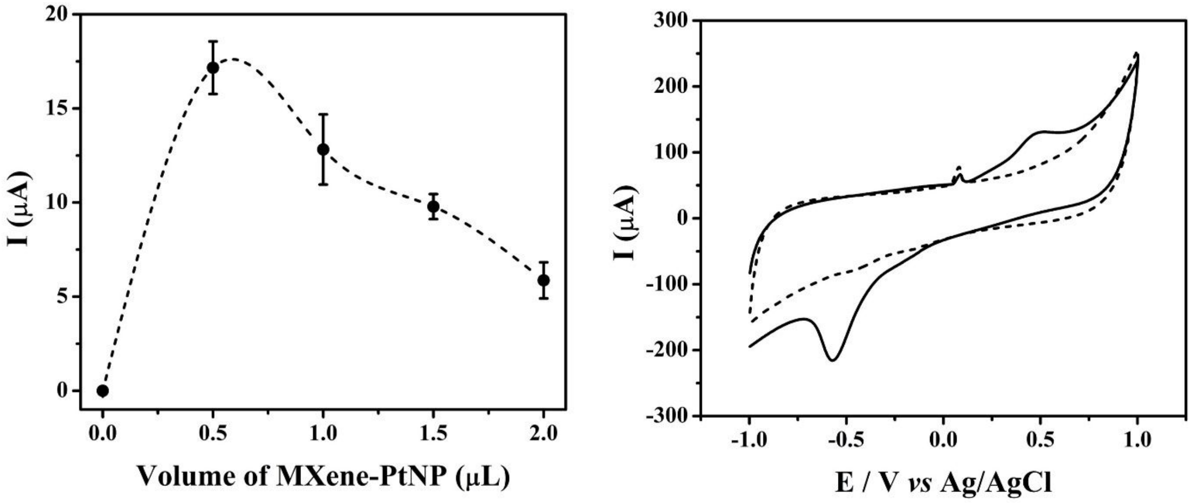

Figure S3: Study of MXene-PtNPs amount on the BC-LSG electrode in the presence of 45 μmol L^{-1} H_2O_2 in KCl 0.1 mol L^{-1}. The dash line corresponds to the VC plot without MXene-PtNPs nanomaterials and black line correspond to LSG electrode modified with 0.5 μL of MXene-PtNPs.

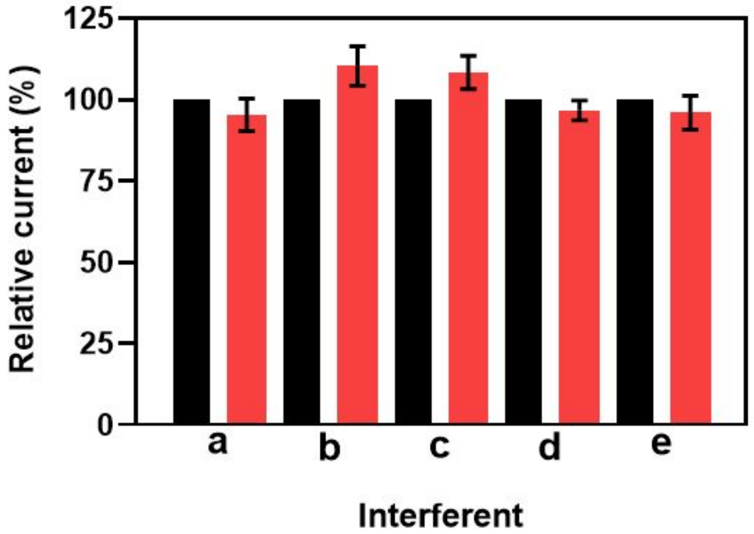

Figure S4: Selectivity studies in the presence of (a) dopamine, (b) uric acid, (c) glucose, (d) citric acid and (e) lactate. The compounds were tested both H_2O_2 (black bar) and in a mix (red bar) with H_2O_2 at a 1:1 ratio (H_2O_2: interferent) at a concentration of 45 μmol L^{-1}.

Table S1: Comparison of the analytical parameters with various methods reported for H_2O_2 detection.

Sensor	Technique	Linear range (μmol L^{-1})	LOD (μmol L^{-1})	Reference

Platinum particles, polyaniline, and MXene	Chronoamperometry	1 − 7	1.0	[1]
Pt/cMIL-68/MoS_2/GCE	Chronoamperometry	0.01-1,830	0.06	[2]
MWCNT PtB composite	Chronoamperometry	0.01-2.0	0.01	[3]
AuNRs/GC	Chronoamperometry	5.0-5,000	1.8	[4]
Mo_2C@MoS_2	EIS	0.23-2,200	0.2	[5]
RGO/Au/Fe_3O_4/Ag nanocomposite	Chronoamperometry	2 − 12	1.43	[6]
$ZnMn_2O_4$@rGO	Chronoamperometry	0.03 − 6000	0.01	[7]
MXene-Co_3O_4/SPEs	LSV	5.0 − 75	0.50	[8]
LSG-PtNPs/rGO	Chronoamperometry	0.5 − 80	0.20	[9]
MXene-PtNPs/BC-LSG	LSV	15 − 95	0.35	This work

References

(1) Neampet, S.; Ruecha, N.; Qin, J.; Wonsawat, W.; Chailapakul, O.; Rodthongkum, N. A Nanocomposite Prepared from Platinum Particles, Polyaniline and a Ti3C2 MXene for Amperometric Sensing of Hydrogen Peroxide and Lactate. *Microchim. Acta* **2019**, *186* (12), 752. https://doi.org/10.1007/s00604-019-3845-3.

(2) Wei, P.; Sun, D.; Niu, Y.; Lu, X.; Zhai, H. Enzyme-Free Electrochemical Sensor for the Determination of Hydrogen Peroxide Secreted from MCF-7 Breast Cancer Cells Using Calcined Indium Metal-Organic Frameworks as Efficient Catalysts. *Electrochim. Acta* **2020**, *359*, 136962. https://doi.org/10.1016/j.electacta.2020.136962.

(3) Abdalla, A.; Jones, W.; Flint, M. S.; Patel, B. A. Bicomponent Composite Electrochemical Sensors for Sustained Monitoring of Hydrogen Peroxide in Breast Cancer Cells. *Electrochim. Acta* **2021**, *398*, 139314. https://doi.org/10.1016/j.electacta.2021.139314.

(4) Maji, S. K. Plasmon-Enhanced Electrochemical Biosensing of Hydrogen Peroxide from Cancer Cells by Gold Nanorods. *ACS Appl. Nano Mater.* **2019**, *2* (11), 7162–7169. https://doi.org/10.1021/acsanm.9b01675.

(5) Shu, Y.; Zhang, L.; Cai, H.; Yang, Y.; Zeng, J.; Ma, D.; Gao, Q. Hierarchical Mo2C@MoS2 Nanorods as Electrochemical Sensors for Highly Sensitive Detection of Hydrogen Peroxide and Cancer Cells. *Sensors Actuators B Chem.* **2020**, *311*, 127863. https://doi.org/10.1016/j.snb.2020.127863.

(6) Heydaryan, K.; Almasi Kashi, M.; Sharifi, N.; Ranjbar-Azad, M. Efficiency Improvement in Non-Enzymatic H 2 O 2 Detection Induced by the Simultaneous Synthesis of Au and Ag Nanoparticles in an RGO/Au/Fe 3 O 4 /Ag Nanocomposite. *New J. Chem.* **2020**, *44* (21), 9037–9045. https://doi.org/10.1039/D0NJ00526F.

(7) Li, Y.; Huan, K.; Deng, D.; Tang, L.; Wang, J.; Luo, L. Facile Synthesis of ZnMn2O4@rGO Microspheres for Ultrasensitive Electrochemical Detection of Hydrogen Peroxide from Human Breast Cancer Cells. *ACS Appl. Mater. Interfaces* **2020**, *12* (3), 3430–3437. https://doi.org/10.1021/acsami.9b19126.

(8) Singh, S.; Numan, A.; Khalid, M.; Bello, I.; Panza, E.; Cinti, S. Facile and Affordable Design of MXene-Co 3 O 4 -Based Nanocomposites for Detection of Hydrogen Peroxide in Cancer Cells: Toward Portable Tool for Cancer Management. *Small* **2023**, *19* (51). https://doi.org/10.1002/smll.202208209.

(9) Bukhari, Q. U. A.; Della Pelle, F.; Alvarez-Diduk, R.; Scroccarello, A.; Nogués, C.; Careta, O.; Compagnone, D.; Merkoci, A. Laser-Assembled Conductive 3D Nanozyme Film-Based Nitrocellulose Sensor for Real-Time Detection of H2O2 Released from Cancer Cells. *Biosens. Bioelectron.* **2024**, *262*, 116544. https://doi.org/10.1016/j.bios.2024.116544.
